# Case report: Altered pre-mRNA splicing caused by intronic variant c.1499 + 1G > A in the *SLC4A4* gene

**DOI:** 10.3389/fped.2022.890147

**Published:** 2022-08-17

**Authors:** Yan Liu, Wenchao Sheng, Jinying Wu, Jie Zheng, Xiufang Zhi, Shuyue Zhang, Chunyu Gu, Detong Guo, Wenhong Wang

**Affiliations:** ^1^Department of Nephrology, Tianjin Children’s Hospital (Tianjin University Children’s Hospital), Tianjin, China; ^2^Graduate College of Tianjin Medical University, Tianjin Medical University, Tianjin, China; ^3^Tianjin Pediatric Research Institute, Tianjin Children’s Hospital (Tianjin University Children’s Hospital), Tianjin, China; ^4^Tianjin Key Laboratory of Birth Defects for Prevention and Treatment, Tianjin, China

**Keywords:** compound heterozygous variants, *SLC4A4*, pre-mRNA, minigene assay, splicing

## Abstract

Proximal renal tubular acidosis (pRTA) with ocular abnormalities is an autosomal recessive disease caused by variants in the Solute Carrier Family 4 Member 4 (*SLC4A4*) gene. Patients present with metabolic acidosis and low plasma bicarbonate concentration (3∼17 mmol/L). In addition, they are often accompanied by ocular abnormalities, intellectual disability, and growth retardation. The patient underwent whole exome sequencing (WES) and bioinformatics analysis of variant pathogenicity in this study. Then, a minigene assay was conducted to analyze the splicing site variant further. Compound heterozygous variants in the *SLC4A4* gene (NM_003759.3), c.145C > T (p.Arg49*) and c.1499 + 1G > A, were detected by WES. The minigene assay showed an mRNA splicing aberration caused by the c.1499 + 1G > A variant. Compared with the wild type, the mutant type caused 4-base insertion between exons 10 and 11 of *SLC4A4* after expression in HEK293 cells. In conclusion, the c.1499 + 1G > A variant in the *SLC4A4* gene may be one of the genetic causes in the patient. Moreover, our study provides the foundation for future gene therapy of such pathogenic variants.

## Introduction

**FIGURE 1:**
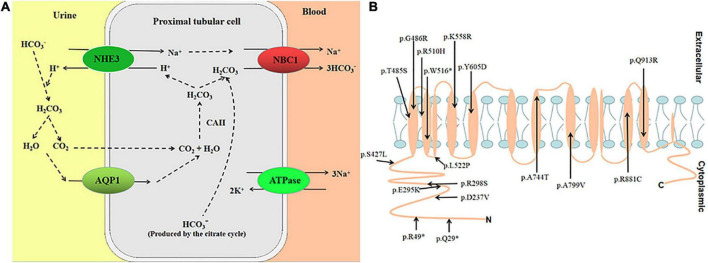
**(A)** Transepithelial HCO_3_^–^ transport is mediated by the coupling of apical Na^+^/H^+^ exchange (NHE3) and basolateral NBC1 functioning as an electrogenic Na^+^-CO_3_^2–^cotransporter with a 1:2 charge transport stoichiometry. **(B)** Putative location of variants in NBC1.

Proximal renal tubular acidosis (pRTA) with ocular abnormalities (OMIM 604278) is an autosomal recessive renal disease. The symptoms include renal tubular acidosis (RTA), ocular abnormalities (glaucoma, band keratopathy, and cataract), intellectual disability, and growth retardation. The disease is caused by variants in the Solute Carrier Family 4 Member 4 (*SLC4A4*) gene. The *SLC4A4* gene spans approximately 450 kb and contains 26 exons (1); it is mapped to chromosome 4q13.3. The human *SLC4A4* gene encodes electrogenic sodium bicarbonate cotransporter 1 (NBC1), mainly expressed in kidney, pancreas, brain, corneal, and other tissues (2). The protein’s primary function is to regulate the secretion and absorption of bicarbonate and regulate intracellular pH (3) (Figure 1A). According to the HGMD variant database, reported studies have found 17 nonsense/missense variants (Figure 1B). Furthermore, the clinical phenotype of patients with nonsense/missense variants is pRTA with ocular abnormalities (4).

In this study, we found a Chinese patient with pRTA and compound heterozygous variants, c.145C > T (p.Arg49*) and c.1499 + 1G > A, in the *SLC4A4* gene (NM_003759.3). Furthermore, *in vitro* functional studies were performed for the splice site variant c.1499 + 1G > A.

## Materials and methods

### Exome sequencing

The whole exome sequencing (WES) of the patient was performed by the KingMed company (Guangzhou, China). Paired-end sequencing was performed in > 95% of target regions, including all coding regions, canonical splice sites, and exon-intron boundaries with reading lengths of 150 bp. Moreover, the sequencing had an average coverage depth of 100-fold. The Burrows-Wheeler Aligner software was used to map raw data reads to the human reference genome GRCh37/hg19. The Genome Analysis Toolkit software was also used to analyze insertion, deletion, and SNP sites. Variant annotations of the KMTD, HGMD, ClinVar, ESP6500, dbSNP, and 1,000 Genomes databases were added with the Annovar software. The classification of variants followed the American College of Medical Genetics and Genomics criteria (ACMG) (5).

### Construction of the *SLC4A4* minigene

Genomic DNA was extracted from the peripheral blood with Blood Genomic DNA Mini Kit (CWBIO, Beijing, China) according to the manufacturer’s instructions. Three fragments of the human *SLC4A4* gene containing exons 9/10/11 of *SLC4A4* and flanking intronic regions were amplified by PCR using a Taq Master Mix dye (CWBIO, Beijing, China) and primers were engineered to have restriction sites (Supplementary material). The PCR products were separated on 1% agarose gel. The gel was purified, purified DNAs were used as templates, and the recombinant plasmid was constructed with pcDNA3.1 and the templates. Then, the constructed recombinant plasmid was sequenced, and verified clones were referred to as the wild type (WT). We obtained mutant type (MUT) clones by Fast Site-Directed Mutagenesis Kit (TIANGEN, Beijing, China). The primers (F-primer: 5′-TCTATTTAATTTCAGCAAATATGTACATACAT-3′, R-pri mer: 5′-TTTGCTGAAATTAAATAGA AGCCTCTCAAAA AC-3′) were used.

### Ribonucleic acid analysis of the overexpressed pcDNA3.1-*SLC4A4* minigene

HEK293T cells from ZQXZBIO (Shanghai, China) were cultured in high-glucose Dulbecco’s modified eagle medium (Gibco, United States) supplemented with 10% fetal bovine serum, 100 U/ml penicillin, and 100 mg/ml streptomycin at 37 and a humidified 5% CO2 incubator. The cells were cultured 24 h before transient transfection in six-well plates. Whereafter, the cells were transfected with the pcDNA3.1-*SLC4A4* minigene (WT, MUT) using the Lipofectamine^®^ 2000 transfection reagent (Thermo Fisher Scientific, United States). Thirty hours after transfection, the cells were harvested and suspended in TRIZOL (TIANGEN, Beijing, China) for total RNA extraction. Then, RNA was reverse-transcribed to cDNA using the FastKing RT Kit (with gDNase) (TIANGEN, Beijing, China) according to the manufacturer’s instructions. The cDNA was amplified using the primer set (F-primer: 5′-GTTCTGTGGTGGACTAATTA-3′, R-primer: 5′-CTGGGTCAGGTGGCACACA-3′). Lastly, RT-PCR products were separated and analyzed on a 1% agarose gel, visualized with DNA ladder and ethidium bromide, gel-purified, and sequenced.

### Bioinformatics analysis

To predict the effect of the variant on pre-mRNA splicing, an *in silico* analysis was performed using Spliceman (6), SpliceAid2 (7), NetGene2 (8), BDGP (9), SROOGLE (10), HEX (11), RBP_map (12), MaxEnt (13), CRYR-SKIP (14), and SpliceAI (15).

## Results

### Clinical presentation

The clinical symptoms of the patient have been previously reported (4). In brief, the patient presented with hypokalemia and general weakness 3 months before hospitalization. Besides, severe acidosis, short stature, epilepsy, encephalopathy, and disturbance of consciousness were shown. Both of the vitreous bodies were cloudy. The head MRI showed high-signal intensity in the bilateral cerebral cortex and symmetrical calcification spots in the bilateral basal ganglia. Therefore, the patient was suspected of having primary pRTA.

### Genetic analysis

The compound heterozygous variants c.145C > T (p.Arg49*) and c.1499 + 1G > A were found in the *SLC4A4* gene (NM_003759.3), but no other variants in the known pRTA pathogenic gene were detected. The variant c.145C > T was inherited from the healthy father, and the variant c.1499 + 1G > A was inherited from the healthy mother. The c.1499 + 1G > A variant had not been included in the HGMD database, which had zero frequency in the 1,000 Genomes Project database.

### Splicing study on *SLC4A4* c.1499 + 1G > A by minigene assay

**FIGURE 2:**
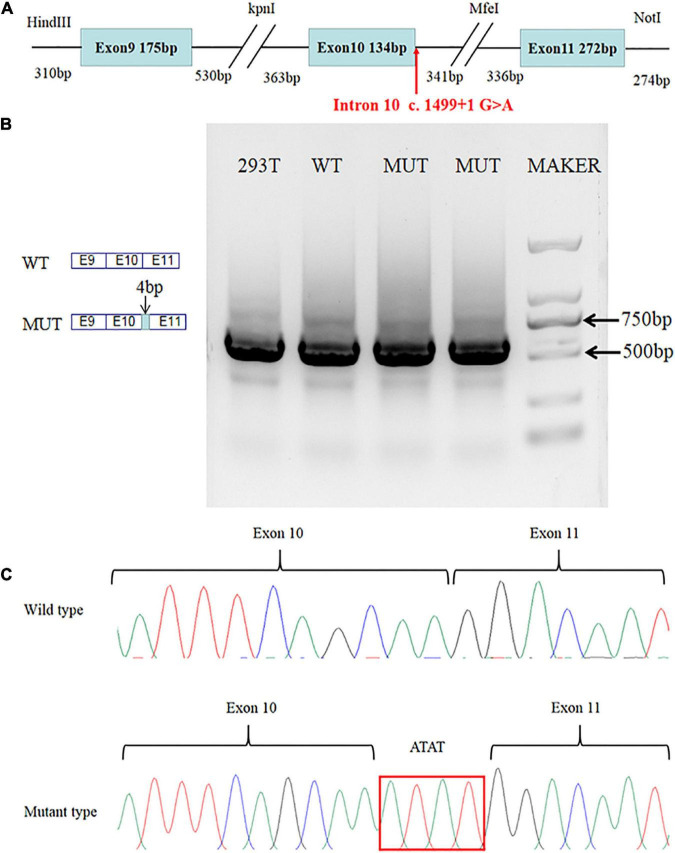
Results of the minigene assay. **(A)** *SLC4A4* minigene structure: schematic diagram of the *SLC4A4* minigene construct containing exon 9, part of intron 9, exon 10, part of intron 10, exon 11, and part of intron 11. **(B)** Agarose gel electrophoresis showed a single band in the WT and one band from the MUT. Moreover, the DNA fragments represented by the two strips might be similar in length. **(C)** The mutant type causes a 4-base insertion between exons 10 and 11 of *SLC4A4* after expression in HEK293 cells.

A minigene analysis was conducted to analyze the splicing site variant further. The *SLC4A4* minigene structure was designed to study the effect of the c.1499 + 1G > A splice-site variant on pre-mRNA splicing (Figure 2A). The results of the RT-PCR products on agarose gel electrophoresis showed a single band in the WT and one band in the MUT. Moreover, the electropherogram showed the DNA fragments represented by the two strips might be similar in length (Figure 2B). The Sanger sequencing showed that WT cDNA-amplified products were consistent with *SLC4A4* exons 9–11. While comparing with *SLC4A4* exons 9–11, we found a 4-base insertion between exons 10 and 11 in MUT cDNA-amplified products (Figure 2C). The minigene analysis showed that the c.1499 + 1G > A variant could abrogate the intron 10 donor splice site. At the same time, the downstream cryptic donor splice site was activated, resulting in the 4 bases (ATAT) of intron 10 getting inserted between exons 10 and 11.

### Bioinformation analysis

Spliceman predicted that the splice site variant would lead to aberrant splicing with an L1 distance of 36,609 and a prediction score of 76%. Variant sequences had significantly lower splice donor scores than wild-type sequences: SROOGLE, 0 vs. 78.15; BDGP, 0 vs. 94; HEX, –4.95 vs. 1.02; MaxEnt, –0.74 vs. 7.44. The SpliceAid2 prediction showed the abnormal position of protein binding to the 5′ splice site (Supplementary material). Moreover, SpliceAI also showed loss of the 5′ splice site. No difference between the WT and the MUT were found with NetGene2 and CRYR-SKIP (Table 1). In addition, the variant transcript sequence was compared with the wild-type transcript sequence with GeneRunner (v6.5.52); the predicted results were splice site variants causing a frameshift.

**TABLE 1:** *In silico* analysis prediction results of the variants in the *SLC4A4* gene.

Variant	Type	Spliceman	SpliceAid2	NetGene2	BDGP	SROOGLE	MaxEnt	HEX	RBP_map	CRYR-SKIP	SpliceAI
c.1499 + 1G > A	Splicing	L1: 36,609 Ranking (L1): 76%	Affecting protein binding	N/A*^a^*	Loss of 5′ splice site	Loss of 5′ splice site	WT: MT = –0.74 :7.44	WT: MT = 1.02 :–4.95	Affecting protein binding	N/A^a^	Loss of splice donor site

^a^No difference between the wild type and the variant type.

## Discussion

In this study, the compound heterozygous variants of the *SLC4A4* gene were found by WES. Then, we further explored the splicing effect of the c.1499 + 1G > A variant. The pre-mRNA splicing mechanism suggests that the spliceosome performs removal of introns. In the process of intron recognition, U1 snRNP recognizes and binds to the AG-GU sequence at the donor splice site. In addition, U2snRNA is combined with a branch point sequence (16). Single nucleotide substitutions at the 5′ or 3′ canonical splice site are the most common splicing variants, leading to activated cryptic splice site, skip exon, or less intron retention (17). The c.1499 + 1G > A variant changes the 5′splice site sequence from 5′-CAAGTAT-3′ to 5′-CAAATAT-3′. It prevents proper recognition of intron 10 by the spliceosome, resulting in a few intron sequence retentions immediately downstream of the variant donor splice site during pre-mRNA splicing. The sequencing results show that the sequence (ATAT) is retained between exons 10 and 11, which caused a frameshift. It is worth noting in the study that the RNA expressed by the *SLC4A4* gene was not detected in the patient′s or his parents’ peripheral blood samples, and kidney tissue cells were unavailable. Therefore, we tried to evaluate the impact of splicing by minigene assay *in vitro*. This method provides a new idea for studying the mechanism of splicing abnormalities when patient samples are not available or genes do not express in peripheral blood (18).

However, in this study, the splicing site information that BDGP predicted was not consistent with *in vitro* experiments (Table 2). SROOGLE showed loss of the 5′ splice site only but did not provide new splice site information. By splicing site analysis, programs can predict whether the splice site is altered, but they are not wholly accurate. In this study, we learn that minigene assay can clarify the mechanism of splicing abnormalities, provide a higher level of pathogenic evidence (PS3) for variants, and improve the pathogenicity classification level of variants. In addition, we further speculated how the cryptic splice site was activated. The prediction of RNA secondary structure by mFold (19) found no difference between the optimal predicted structures of the wild type and the mutant type, but the 5′ donor splice site of intron 10 and cryptic splice sites were exposed in the same hairpin loop region (Supplementary material). Combining the results of the above minigene assay and SpliceAid2 predictions, we consider that one of the reasons for the activation of the cryptic splice site may be that the c.1499 + 1G > A variant makes the normal donor splice site disappear. The cryptic splice site in the hairpin loop becomes particularly exposed, and the spliceosome recognizes cryptic splice sites leading to splicing abnormalities.

**TABLE 2:** Results of BDGP and minigene assay.

	Donor site predictions for *SLC4A4*		Acceptor site predictions for *SLC4A4*	
	Score	Splice site sequence	Score	Splice site sequence
Wild type	0.94	tcagcaaGTatgtac	0.98	ttttctttattttAGggcgtgttgga
	0.42	caattagGTagtcac		
Mutant type	0.42	caattagGTagtcac	0.98	ttttctttattttAGggcgtgttgga
Minigene assay	–^a^	tcagcaaatatGTacatacat	–^a^	ttttctttattttAGggcgtgttgga

^a^No data.

The red word represents c.1499 + 1G > A; the capital letters are splice sites.

In conclusion, the c.1499 + 1G > A in the *SLC4A4* gene is a null variant (PVS1). *In vitro* or *in vivo* functional studies on the c.1499 + 1G > A variant demonstrates that the variant has a damaging effect (PS3). c.1499 + 1G > A is absent from controls in 1,000 Genomes Project (PM2). For recessive disorders, they are detected in trans with a pathogenic variant (PM3). Multiple lines of computational evidence support a deleterious effect on the gene (PP3). According to the ACMG guidelines, the variant is classified as “pathogenic” based on the above evidence. In addition, the clinical manifestation of pRTA with ocular abnormalities is severe, and the prognosis is poor. Prenatal diagnosis and genetic counseling are vital for such disease. Early identification of pathogenic variant is the basis of prenatal diagnosis and genetic counseling. Our study is of great significance to prenatal diagnosis and prevention of birth of children with the disease. Moreover, the therapeutic potential of antisense oligonucleotides (ASOs) for correcting splicing defects that cause genetic diseases has been investigated. It was reported that ASOs could improve the abnormal splicing effect of the c.6937 + 594T > G variant in the *BRCA2* gene to a certain extent (20). Another study showed that ASOs blocked the aberrant splicing created by *CFTR* c.3718-2477C > T mutation, redirected the splicing to the correct splicing site, and restored chloride secretion in bronchial epithelial cells of patients with cystic fibrosis (21). Our study provides the basis for future gene therapy of such pathogenic variants.

## Conclusion

In conclusion, *SLC4A4* variants, including c.1499 + 1G > A and c.145C > T (p.Arg49*), may be the genetic basis of the patient. Moreover, the splicing abnormality is verified *in vitro*, and this discovery will contribute to accurate diagnosis. Further functional studies may help reveal the potential mechanism of *SLC4A4* gene variants causing pRTA with ocular abnormalities and provide possible gene therapy in the future.

## Data availability statement

The original contributions presented in this study are included in the article/Supplementary material, further inquiries can be directed to the corresponding author.

## Ethics statement

The studies involving human participants were reviewed and approved by the Ethical Committee of Tianjin Children’s Hospital. Written informed consent was obtained from the minor(s)’ legal guardian/next of kin for the publication of any potentially identifiable images or data included in this article.

## Author contributions

YL, WS, and WW designed the experiments. YL, WS, JW, JZ, and SZ performed the experiments. WS wrote the manuscript. WW, SZ, XZ, DG, JZ, and CG provided revisions and edits of the manuscript. All authors commented on previous versions of the manuscript and read and approved the final version of the manuscript.
