# Supplementary material for: Case report: Altered pre-mRNA splicing caused by intronic variant c.1499 + 1G > A in the SLC4A4 gene
Source: Front Pediatr. 2022 Aug 17;10:890147. doi: 10.3389/fped.2022.890147 (PMC9428394; doi:10.3389/fped.2022.890147)
Supplement: Supplementary file 2 [file Data_Sheet_2.ZIP › Raw data_addition files/Table Minigene primer design.docx]

Table :Minigene primer design

| PCR Product | Primer Sequence | Including Exon | Restriction Enzymes |
| --- | --- | --- | --- |
| Fragment A | F:5’-CCCAAGCTTCTTGCATATTAGGACGTTT-3’  R:5’-CGGGGTACCTTAATGAGAATCAGGTCCAC-3’ | Exon 9 | HindIII kpnI |
| Fragment B | F:5’-CGGGGTACCTTGCCACCAAAAGTACCAA-3’  R:5’-CCGCAATTGGTACTCCCCATTAGCCAAA-3’ | Exon 10 | KpnI MfeI |
| Fragment C | F:5’-CCGCAATTGTAGGTACCAGTGCTATGCTT-3’  R:5’-ATTTGCGGCCGCATGAATTCCTTGCAAATCCCA-3’ | Exon 11 | MfeI NotI |
