# Supplementary figures and images for: Case report: Altered pre-mRNA splicing caused by intronic variant c.1499 + 1G > A in the SLC4A4 gene
Source: Front Pediatr. 2022 Aug 17;10:890147. doi: 10.3389/fped.2022.890147 (PMC9428394; doi:10.3389/fped.2022.890147)

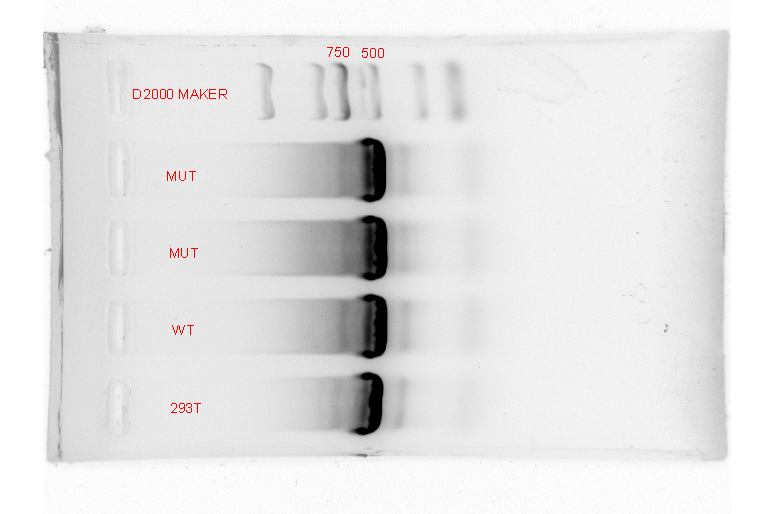

Supplement: Supplementary file 2 [file Data_Sheet_2.ZIP › Raw data_addition files/RNA analysis of the Overexpressed pcDNA3.1-SLC4A4 gene Minigene/01-scans of the entire original gels.tif]

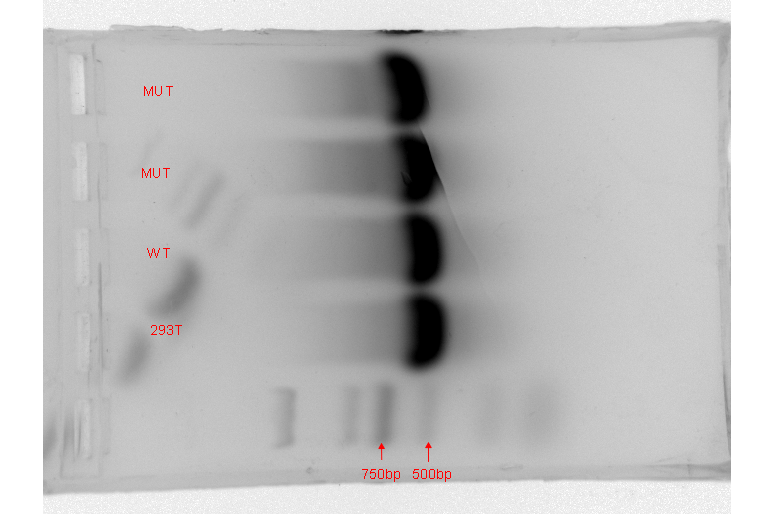

Supplement: Supplementary file 2 [file Data_Sheet_2.ZIP › Raw data_addition files/RNA analysis of the Overexpressed pcDNA3.1-SLC4A4 gene Minigene/02-scans of the entire original gels.tif]

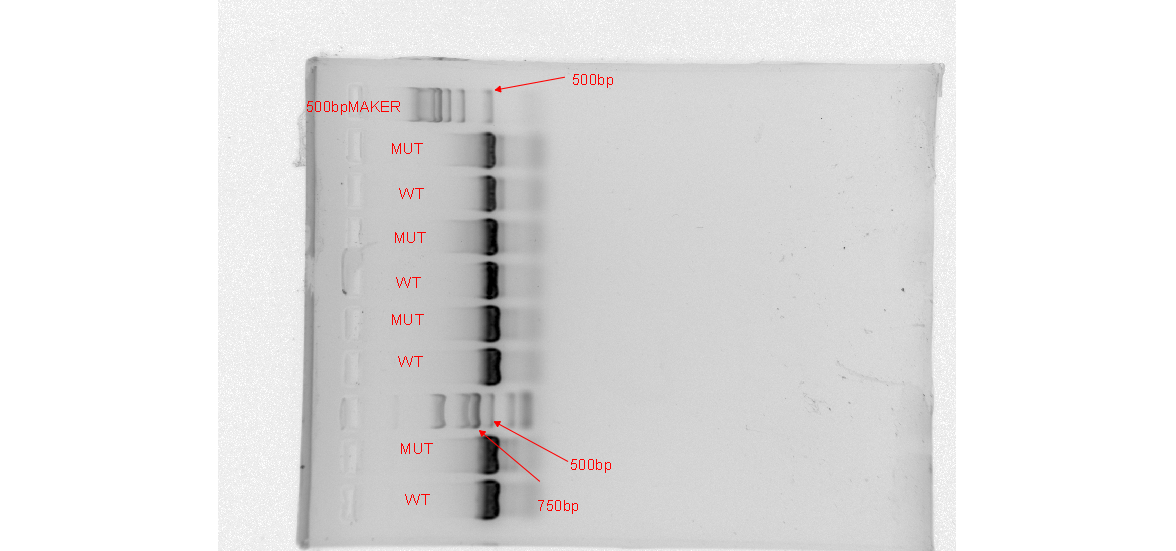

Supplement: Supplementary file 2 [file Data_Sheet_2.ZIP › Raw data_addition files/RNA analysis of the Overexpressed pcDNA3.1-SLC4A4 gene Minigene/03-scans of the entire original gels.tif]
