# Supplementary material for: Case report: Altered pre-mRNA splicing caused by intronic variant c.1499 + 1G > A in the SLC4A4 gene
Source: Front Pediatr. 2022 Aug 17;10:890147. doi: 10.3389/fped.2022.890147 (PMC9428394; doi:10.3389/fped.2022.890147)
Supplement: Supplementary file 2 [file Data_Sheet_2.ZIP › Raw data_addition files/mFOLD.zip!/mFOLD/mFold-WT/22Jul16-06-53-36.pdf]

Output of boxplot_ng (©)
mfold_util 4.7

Created Sat Jul 16 06:54:04 2022

Fold of SLC4A4−minigene−WT at 37 C.

δG in Plot File = 12.0 kcal/mol

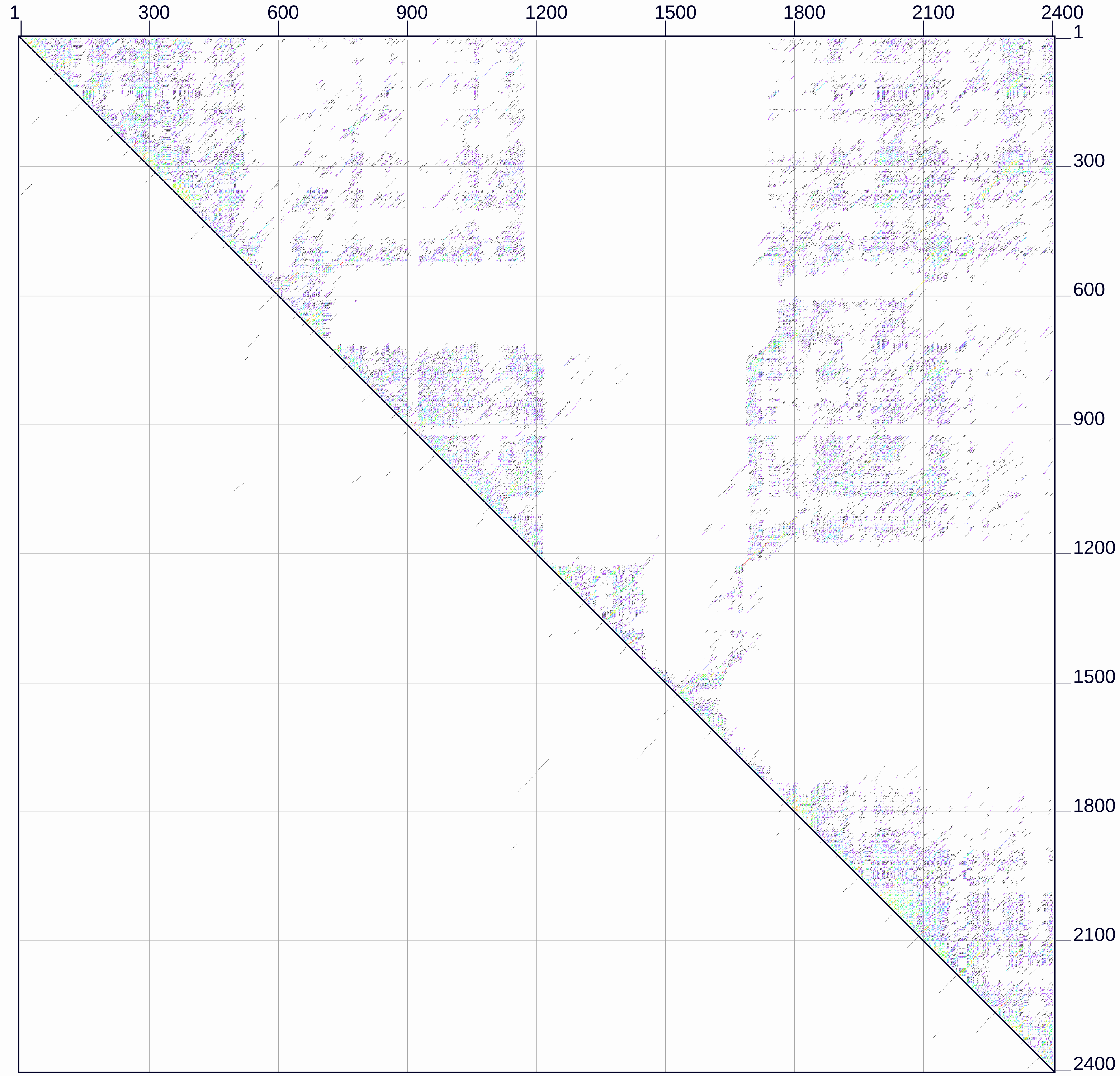

Lower Triangle: Optimal Energy
Upper Triangle Base Pairs Plotted: 160300

Optimal Energy = -578.3 kcal/mol
-578.3 < Energy <= -576.6 kcal/mol
-576.6 < Energy <= -574.9 kcal/mol
-574.9 < Energy <= -573.2 kcal/mol
-573.2 < Energy <= -571.4 kcal/mol
-571.4 < Energy <= -569.7 kcal/mol
-569.7 < Energy <= -568.0 kcal/mol
-568.0 < Energy <= -566.3 kcal/mol
